# Medical research agency: 5 years of reshaping the clinical trials ecosystem in Poland

**DOI:** 10.1016/j.lanepe.2024.101175

**Published:** 2024-12-11

**Authors:** Diana Kitala, Katarzyna Kaczmarska, Joanna Kornacka, Krzysztof Górski, Elżbieta Bylina, Karolina Nowak, Rafał Staszewski, Zuzanna Nowak-Życzyńska, Wojciech Fendler

**Affiliations:** a Medical Research Agency, Chmielna 69, 00-801, Warsaw, Poland; b Poznan University of Medical Sciences, Fredry 10, 61-701, Poznań, Poland; c Department of Biostatistics and Translational Medicine, Medical University of Lodz, Mazowiecka 15, 92-215, Lodz, Poland; d Department of Radiation Oncology, Dana-Farber Cancer Institute, 4 Blackfan, 00-215, Boston, MA, USA

Total global investment in biomedical and health research is constantly increasing, from USD 240 billion in 2009 to approximately USD 300 billion in 2023 and governments invariably remain the main source of biomedical research funding.^1^ Still, the rudimentary question remains the same–are allocation patterns appropriate? Underfunding or overfunding of particular diseases has been a perpetual topic of debate in many funding bodies worldwide.^2^ In Poland, these global challenges are similarly reflected in the allocation of funding for research in various therapeutic areas, with a growing need for a more equitable and efficient model. Within cancer research, haematological and lung cancers were associated with the lowest investment per mortality burden.^3^ A proper financing model should consider prevalence, mortality, clinical demands, existing therapies and carefully assess existing financing providers. For instance, despite the International Cancer Research Partnership, there are 4693 organisations providing cancer research funding, 21% of which are based in Europe.^3^ The WHO has urged the need to addressing the underrepresentation of children in clinical trials,^4^ which makes it another issue requiring immediate action to promote the presence of paediatric populations.

The Medical Research Agency is a state agency, established in 2019, addressed the lack of a central body managing clinical studies in Poland. According to the Act of February 21, 2019, on the Medical Research Agency,^5^ the core functions of the MRA include funding research in medical and health sciences, focusing on clinical, observational, and epidemiological studies, as well as medical experiments. The Agency was also entrusted with the task of designing the Governmental Plan for the Development of the Biomedical Sector in Poland for 2022–2031.^6^ Additionally, the MRA engages in issuing expert opinions and reports and initiating independent research and development projects.

Throughout 5 years of its activities, the MRA successfully achieved significant milestones. The total number of signed co-financing agreements reached 315, worth over USD 1.03 billion (PLN 4.3 billion), averaging USD 3.3 million (PLN 13.7 million) per project. These non-commercial clinical trial concentrate on three primary research areas: cardiovascular diseases, oncology and haematology, and neurology and psychiatry. As part of the signed agreements, over 51,000 patients, including more than 13,000 with rare diseases, will be enrolled in the studies. This data highlights the MRA's significant contribution to advancing clinical research in critical medical fields and underscores its commitment to addressing unmet medical needs, particularly in the areas of rare diseases and paediatric care. The development of healthcare infrastructure plays a crucial role in ensuring access to high-quality healthcare services and improving health outcomes.^7^^,^^8^ To enable the MRA to fulfil its tasks, it was necessary to create a favourable environment for investigators to initiate non-commercial clinical trials and increase access to innovative research and medical infrastructure. The idea behind creating dedicated Clinical Trial Support Centres (CTSCs) is to harness the potential of Polish facilities engaged in clinical trials.

In 2020 and 2021, the Agency announced two editions of the call for proposals for establishing and developing CTSCs and one focused on oncology and haemato-oncology CTSCs (OncoCTSCs; Fig. 1). The CTSCs, part of the Polish Clinical Trials Network, are outpatient centres (some offering hospitalization) improving access to modern clinical trials in Poland. Recognizing the growing importance of digital medicine in modern healthcare delivery, the MRA also established Regional Digital Medicine Centres (RDMCs). These centres will integrate healthcare data, including hospital systems and clinical trial information, ensuring efficient analysis while maintaining data security. RDMCs will support clinical trials and hospital care, positioning Poland as a leader in digital health solutions.

**Fig. 1:**
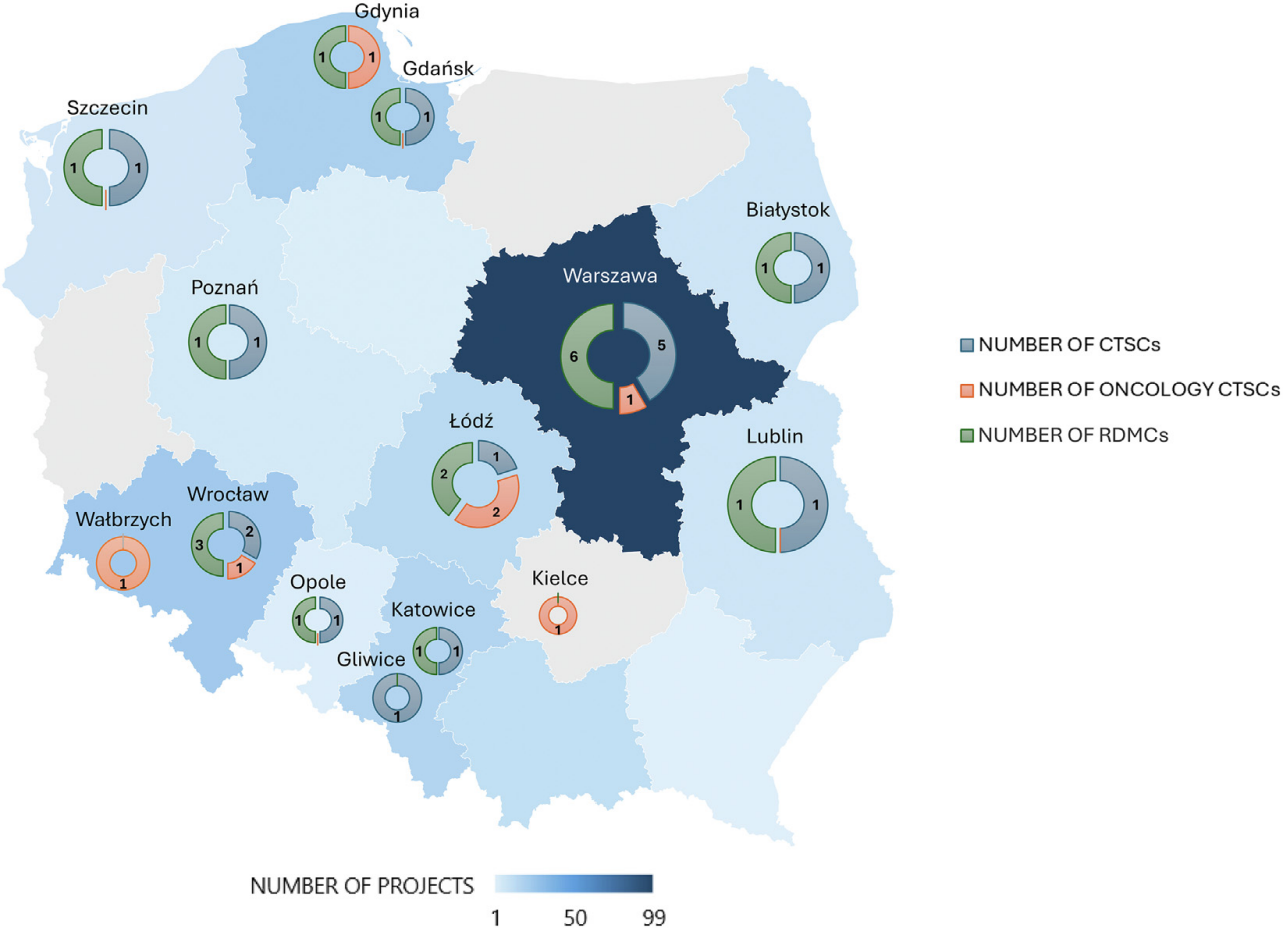
Geographic distribution of Clinical Trial Support Centres (CTSCs), Oncologic Clinical Trial Support Centres (OncoCTSCs) and Regional Digital Medicine Centres (RDMCs; detailed description of Units in Supplementary Table S1). The number of specialized centres funded through four subsequent calls for CTSCs, OncoCTSCs and RDMCs is provided for calls concluded by the end of 2024. Two new calls are ongoing under the EU funding mechanism of Poland's National Recovery and Resilience Plan with a tentative number of at least 10 new CTSCs to be formed and at least 18 gaining additional funds for expansion and broadening of capacity. The colour of the administrative regions (voivodeships) represents the number of projects (clinical trials and commercial R&D initiatives) contracted by institutions based in those areas. For projects conducted under consortium agreements, the site of the study Sponsor/Consortium leader was used for this visualization.

Since 2021, the MRA has launched a series of training courses aimed at improving the clinical trials landscape. These sessions have been attended by a total of 7731 participants and 287 patients. The courses include both open and specialized sessions tailored for doctors, researchers, clinical trial teams, Bioethics Committee members, and individuals interested in clinical trials. Additionally, 364 patients and patient organization members, as well as 794 medical students, enrolled in courses designed for them. The flagship educational initiative of the MRA is the training programme conducted by Harvard Medical School Postgraduate Medical Education—the Polish Clinical Scholar Research Training (P-CSRT). This programme, part of MRA's Educational Strategy for 2023–2027, aims to train 500 Polish researchers in study design, data analysis, and scientific publication, enhancing their ability to plan clinical trials and publish results. The first edition concluded in October 2024.

The first 5 years of MRA's existence have already reshaped the clinical trials ecosystem in Poland. The Agency's support in developing the PCTN enables high quality clinical research to be conducted in Poland and enables Polish scientists to participate in global research. Competitive, substantial funding, coupled with the ever-growing potential of clinical and research personnel, together with state-of-the-art CTSCs create a perfect storm for top-tier clinical trials including ones conducted jointly with foreign partners.

## Contributors

DK literature search, data collection, data analysis, methodology, KK-literature search, data collection, data analysis, methodology, JK–literature search, data collection, data analysis, methodology, KG-data collection, EB–data collection, KN–data collection, writing original draft, RS–data collection, study conceptualization, ZNZ–writing review and editing, data curation, methodology, WF–study conceptualization, data curation, methodology, writing review and editing.

## Editor note

The Lancet Group takes a neutral position with respect to territorial claims in published maps and institutional affiliations.

## Declaration of interests

None of the Authors have any conflict of interest to disclose.
