# Supplementary material for: Medical research agency: 5 years of reshaping the clinical trials ecosystem in Poland
Source: Lancet Reg Health Eur. 2024 Dec 11;48:101175. doi: 10.1016/j.lanepe.2024.101175 (PMC11699205; doi:10.1016/j.lanepe.2024.101175)
Supplement: Supplementary Table S1 [file mmc1.docx]

**Supplementary Table 1. Overview of CTSCs, Oncological CTSCs, and RDMCs Institutions in Major Polish Cities.**

| **City** | **Scientific Centre** | **CTSCs** | **Oncological CTSCs** | **RDMCs** |
| --- | --- | --- | --- | --- |
| GDAŃSK | Medical University of Gdańsk | X |  | X |
| GDYNIA | Pomeranian Hospitals LLC |  | X | X |
| BIAŁYSTOK | Medical University of Bialystok | X |  | X |
| WARSZAWA | National Institute of Cardiology Of Stefan Cardinal Wyszyński - National Research Institute | X |  | X |
|  | Central Clinical Hospital of the Ministry of Interior and Administration in Warsaw | X |  | X |
|  | "Monument - Children's Health Centre" Institute in Warsaw | X |  | X |
|  | Military Medical Institute in Warsaw | X |  | X |
|  | National Geriatrics, Rheumatology and Rehabilitation Institute, name of prof· Eleonora Reicher in Warsaw | X |  | X |
|  | Institute of Haematology and Transfusion Medicine in Warsaw |  | X |  |
|  | Maria Sklodowska-Curie National Research Institute of Oncology in Warsaw |  |  | X |
| LUBLIN | Medical University of Lublin | X |  | X |
| ŁÓDŹ | Medical University of Lodz | X |  | X |
|  | The Polish Mother's Health Centre Institute in Lodz |  | X |  |
|  | Voivodeship Multi-Specialist Centre for Oncology and Traumatology in Lodz |  | X | X |
| KIELCE | Holy Cross Cancer Centre |  | X |  |
| KATOWICE | Medical University of Silesia in Katowice | X |  | X |
| GLIWICE | Maria Sklodowska-Curie Institute – Oncology Centre (MSCI), branch in Gliwice | X |  |  |
| OPOLE | University of Opole | X |  | X |
| WAŁBRZYCH | Dr Alfred Sokolowski Specialist Hospital in Walbrzych |  | X |  |
| WROCŁAW | Wroclaw Medical University | X |  | X |
|  | 4 Military Clinical Hospital with Polyclinic SPZOZ in Wroclaw | X |  | X |
|  | Lower Silesian Oncology Centre in Wroclaw |  | X | X |
| POZNAŃ | Poznan University of Medical Sciences | X |  | X |
| SZCZECIN | Pomeranian Medical University in Szczecin | X |  | X |
